# Characteristics and outcomes of hospital-acquired and community-acquired peritonitis in patients on peritoneal dialysis: a retrospective cohort study

**DOI:** 10.1007/s40620-023-01597-w

**Published:** 2023-03-13

**Authors:** Chau Wei Ling, Kamal Sud, Gregory Peterson, Judith Fethney, Connie Van, Rahul Patel, Syed Tabish Razi Zaidi, Ronald Castelino

**Affiliations:** 1 Faculty of Medicine and Health, The University of Sydney, Sydney, NSW Australia; 2 Department of Renal Medicine, Nepean Kidney Research Centre, Nepean Hospital, Sydney, NSW Australia; 3 Departments of Renal Medicine, Nepean, Blacktown and Westmead Hospitals, Sydney, NSW Australia; 4 Peritoneal Dialysis Unit, Regional Dialysis Centre, Blacktown Hospital, Sydney, NSW Australia; 5 School of Pharmacy and Pharmacology, University of Tasmania, Hobart, TAS Australia; 6 Faculty of Health, University of Canberra, Australian Capital Territory, Bruce, Australia; 7 Faculty of Medicine and Health, Susan Wakil School of Nursing and Midwifery, The University of Sydney, Sydney, Australia; 8 Professional Services Unit, HPS Pharmacies, EBOS Group, Docklands, VIC Australia; 9 Department of Pharmacy, Blacktown Hospital, Blacktown, NSW Australia

**Keywords:** Hospital-acquired, Outcomes, Peritoneal dialysis, Peritonitis

## Abstract

**Background:**

Peritonitis remains a significant complication of peritoneal dialysis. However, there is limited information on the clinical characteristics and outcomes of hospital-acquired peritonitis compared to community-acquired peritonitis in patients undergoing peritoneal dialysis. Furthermore, the microbiology and outcomes of community-acquired peritonitis may vary from hospital-acquired peritonitis. Therefore, the aim was to gather and analyse data to address this gap.

**Methods:**

Retrospective review of the medical records of all adult patients on peritoneal dialysis within the peritoneal dialysis units in four university teaching hospitals in Sydney, Australia, who developed peritonitis between January 2010 and November 2020. We compared the clinical characteristics, microbiology and outcomes of community-acquired peritonitis and hospital-acquired peritonitis. Community acquired peritonitis was defined as the development of peritonitis in the outpatient setting. Hospital-acquired peritonitis was defined as: (1) developed peritonitis anytime during hospitalisation for any medical condition other than peritonitis, (2) diagnosed with peritonitis within 7 days of hospital discharge and developed symptoms of peritonitis within 3 days of the hospital discharge.

**Results:**

Overall, 904 episodes of peritoneal dialysis-associated peritonitis were identified in 472 patients, of which 84 (9.3%) episodes were hospital-acquired. Patients with hospital-acquired peritonitis had lower mean serum albumin levels compared to those with community-acquired peritonitis(22.95 g/L vs. 25.76 g/L, *p* = 0.002). At the time of diagnosis, lower median peritoneal effluent leucocyte and polymorph counts were observed with hospital-acquired peritonitis compared to community-acquired peritonitis (1236.00/mm^3^ vs. 3183.50/mm^3^, *p* < 0.01 and 1037.00/mm^3^ vs. 2800.00/mm^3^, *p* < 0.01, respectively). Higher proportions of peritonitis due to *Pseudomonas spp.* (9.5% vs. 3.7%, *p* = 0.020) and *vancomycin-resistant Enterococcus* (2.4% vs. 0.0%, *p* = 0.009), lower rates of complete cure (39.3% vs. 61.7%, *p* < 0.001), higher rates of refractory peritonitis (39.3% vs. 16.4%, *p* < 0.001) and higher all-cause mortality within 30 days of peritonitis diagnosis (28.6% vs. 3.3%, *p* < 0.001) were observed in the hospital-acquired peritonitis group compared to the community-acquired peritonitis group, respectively.

**Conclusions:**

Despite having lower peritoneal dialysis effluent leucocyte counts at the time of diagnosis, patients with hospital-acquired peritonitis had poorer outcomes, including lower rates of complete cure, higher rates of refractory peritonitis and higher rates of all-cause mortality within 30 days of diagnosis, compared to those with community-acquired peritonitis.

**Graphic abstract:**

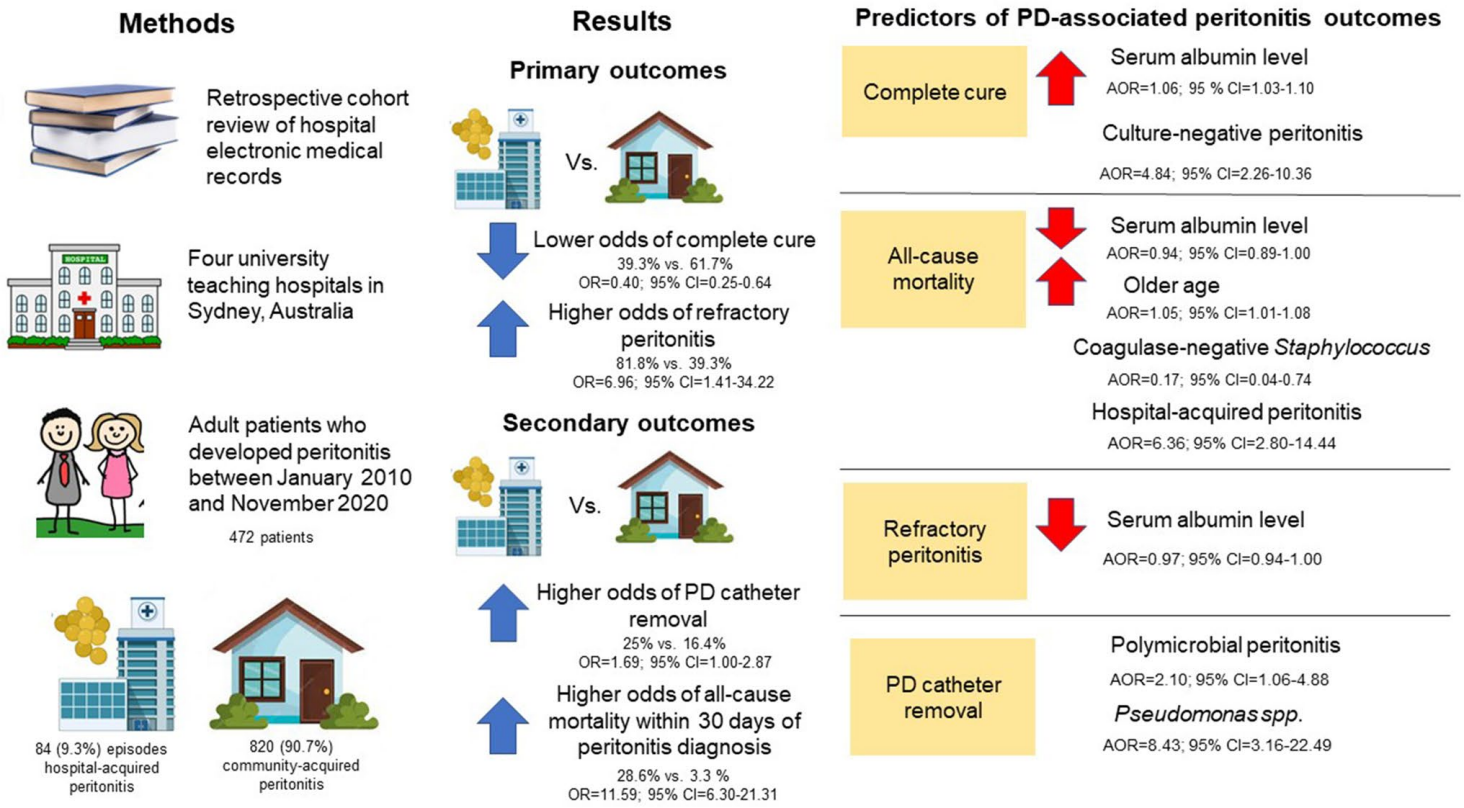

**Supplementary Information:**

The online version contains supplementary material available at 10.1007/s40620-023-01597-w.

## Introduction

Peritonitis remains a significant complication of peritoneal dialysis (PD) and an important cause of permanent transfer to haemodialysis [1]. Hospitalised patients on PD are more susceptible to peritonitis due to multiple risk factors [2–5], which include touch contamination by untrained hospital personnel accessing the PD-catheter, constipation from immobilisation, invasive diagnostics and therapeutic procedures (i.e. gastroscopy), malnutrition during hospitalisation, and recent antibiotic therapy.

Most research on the clinical characteristics and outcomes in patients with hospital-acquired peritonitis (HaP) is limited to small retrospective single-centre studies conducted over 15 years ago [6, 7]. Of note, a previous single-centre retrospective cohort study [7] reported no significant differences in the causative organisms and outcomes between the community-acquired peritonitis (CaP) and HaP groups. In contrast, Troidle et al*.* [6] found a higher prevalence of peritonitis caused by *Enterococcus spp.*, *Candida spp.* and Gram-negative organisms and prolonged duration of hospital stay in the HaP group. However, these studies may now be outdated as microbiological evolution has likely occurred over the years, in addition to changes in PD connectology, patient comorbidities, and the types of invasive procedures patients undergo during hospitalisation.

Due to the lack of data, there are no guidelines on preventing and treating patients with HaP. Given that causative organisms may be different, and the poorer outcomes of patients with HaP, it may not be appropriate to consider adopting the same treatment approaches used for patients with CaP in patients with HaP. Previously, Bunke et al. [8] suggested that the treatment of peritonitis should not be a ‘one-size-fits-all” approach given that the outcomes of each peritonitis vary based on causative organisms, suggesting the need to tailor the choice of empirical antibiotics based on unit-specific profiles of organisms causing HaP and their sensitivity patterns. Therefore, more research is required to evaluate whether CaP and HaP warrant different preventive strategies or therapeutic approaches based on the organism-specific rates in the current era. This study aimed to compare the spectrum of organisms, clinical characteristics, and outcomes between CaP and HaP. The predictors associated with the outcomes of CaP and HaP will also be discussed.

## Methods

### Study design and setting

A retrospective cohort review of hospital electronic medical records within the PD units in four university teaching hospitals in Sydney, Australia, managed by a single PD service. Adult patients who developed peritonitis between January 2010 and November 2020 were included in the review. All patients on automated cycler-delivered PD (APD) when hospitalised were converted to manual PD exchanges (CAPD) during the hospital stay. This study was approved by the Institutional Human Research Ethics Committee (2020/PID03105).

### Inclusion criteria

Patients ≥ 18 years old on PD and diagnosed with peritonitis based on the International Society for Peritoneal Dialysis (ISPD) peritonitis guidelines criteria [9].

### Study definitions

CaP was defined as the development of peritonitis outside the hospital and more than 3 days post-hospital discharge. HaP was defined as: (1) developed peritonitis anytime during hospitalisation for any medical condition other than peritonitis, or (2) diagnosed with peritonitis within 7 days of hospital discharge and developed symptoms of peritonitis within 3 days of the recent hospital discharge [6]. Hospitalised patients with CaP who developed another peritonitis episode during that hospitalisation (Ca-t-HaP) were classified as a separate group: patients with dialysate culture that yielded a different organism or uptrend in the dialysate white cell counts after initial improvement with antibiotics were classified in this group.

Complete cure was defined as the resolution of the peritonitis episode with antibiotics alone without relapse or recurrence within 4 weeks of antibiotic completion, the need for PD-catheter removal, the need for transfer to haemodialysis for ≥ 30 days, or death [9]. Relapsing peritonitis was defined as a peritonitis episode within 4 weeks of antibiotic completion with the same organism and/or a culture-negative episode [9]. Recurrent peritonitis was defined as a peritonitis episode with a different organism within 4 weeks of antibiotic completion [9]. Refractory peritonitis was defined as dialysate failing to clear after 5 days of appropriate antibiotic treatment [9]. All-cause mortality was defined as any death due to any cause within 30 days of a peritonitis diagnosis.

### Data collection

The data collected included: patient demographics, comorbidities at the time of peritonitis (chronic lung disease, coronary artery disease, peripheral vascular disease, cerebrovascular disease, diabetes mellitus), the primary cause of end-stage kidney disease, PD modality, smoking status at renal replacement therapy entry, serum albumin level at the time of diagnosis of peritonitis, PD fluid cytology and microbiology results at peritonitis diagnosis, date of peritonitis diagnosis, reasons for hospital admission and types of invasive procedures leading to HaP, and length of hospital stay prior to the development of HaP and after developing HaP. The primary outcomes examined were complete cure, refractory, relapsing, and recurrent peritonitis. Secondary outcomes were PD-catheter removal and all-cause mortality within 30 days of diagnosis of peritonitis, and identification of the predictors associated with these outcomes. Data was collected through electronic medical records and the Renal Information System Catalogue (RISC) database that records information on all patients on dialysis.

### Statistical analyses

Results are presented as frequencies and percentages for categorical variables, continuous variables as mean ± standard deviation for normally distributed variables and median (interquartile range [IQR]) for non-normally distributed continuous variables. Comparison between groups (CaP vs. HaP) and (HaP vs. Ca-t-HaP) was conducted using a chi-square test or Fisher exact test for categorical variables, while continuous variables were compared using the Student *t*-test and Mann–Whitney *U* test, as appropriate. A generalised estimating equation (GEE) with binomial distribution was used to determine predictors potentially associated with outcomes with *p*-value < 0.05 (Table 3) in the CaP and HaP groups to account for the multiple events of peritonitis episodes within individuals across the study period. All covariates with a *p*-value < 0.2 in univariate analysis were added to the multivariable GEE model. Statistical analyses were performed using SPSS (IBM Corp., Released 2020, IBM SPSS Statistics for Windows, version 27, Armonk, NY, USA). A *p*-value < 0.05 was considered statistically significant.

## Results

### Patient characteristics

**Fig. 1:**
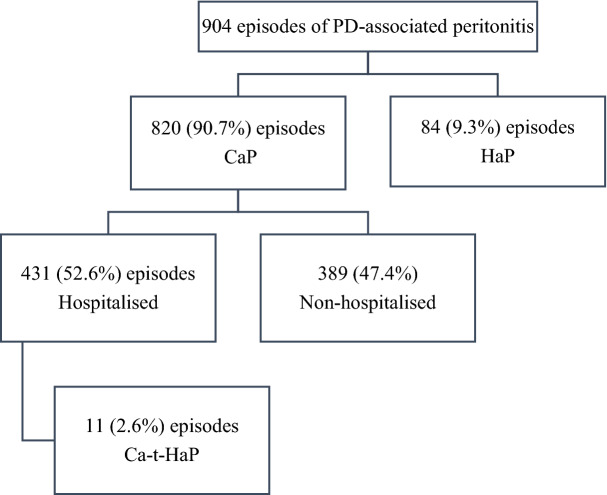
Flow diagram that summarises the breakdown of PD-associated peritonitis episodes. *CaP* Community-acquired peritonitis, *HaP* Hospital-acquired peritonitis, *Ca-t-HaP* Community-acquired then-developed hospital-acquired peritonitis, *PD* Peritoneal dialysis

A total of 904 episodes of PD-associated peritonitis were identified in 472 patients during the study period. Of these, 820 (90.7%) episodes were CaP and 84 (9.3%) episodes were HaP. Among the CaP group, 431 (52.6%) episodes required hospitalisation, while 389 (47.4%) were treated in the outpatient setting. Of the CaP group that required hospitalisation, 11 (2.6%) episodes further developed HaP during hospitalisation (Fig. 1). The median (IQR) dialysis vintage duration of CaP was 22.61 (11.02–42.55) months, while HaP was 19.70 (8.19–40.70) months. The baseline characteristics of the patients at the time of peritonitis are summarised in Table 1.

**Table 1:** Baseline characteristics of PD patients with peritonitis

Characteristics	All patients (*n* = 472)
Male sex, *n* (%)	293 (62.1%)
Age (years), mean ± SD	62.4 ± 14.93
Racial origin,* n* (%)	
White	221 (46.8%)
Asian	169 (35.8%)
Maori-Pacific Islanders, Aboriginal and Torres Strait Islanders	59 (12.5%)
Other	23 (4.9%)
Cause of ESKD, *n* (%)	
Diabetic nephropathy	214 (45.3%)
Glomerulonephritis	90 (19.1%)
Hypertension	67 (14.2%)
Other	67 (14.2%)
Polycystic kidney disease	24 (5.1%)
Unknown	10 (2.1%)
PD modality^a^, *n* (%)	
CAPD	815 (90.2%)
APD	89 (9.8%)
Comorbidities at peritonitis diagnosis, *n* (%)	
Diabetes mellitus	241 (51.1%)
Coronary artery disease	162 (34.3%)
Cerebrovascular disease	53 (11.2%)
Chronic lung disease	50 (10.6%)
Peripheral vascular disease	28 (5.9%)
Current smoker	29 (6.1%)
No. of peritonitis episodes, *n* (%)	
1	254 (53.8%)
2	115 (24.4%)
≥ 3	103 (21.8%)

*APD* automated peritoneal dialysis, *CAPD* continuous ambulatory peritoneal dialysis, *ESKD* end-stage kidney disease, *PD* peritoneal dialysis, *SD* standard deviation

^a^All hospitalised patients received CAPD during their hospital stay

### Characteristics of hospital-acquired peritonitis

**Fig. 2:**
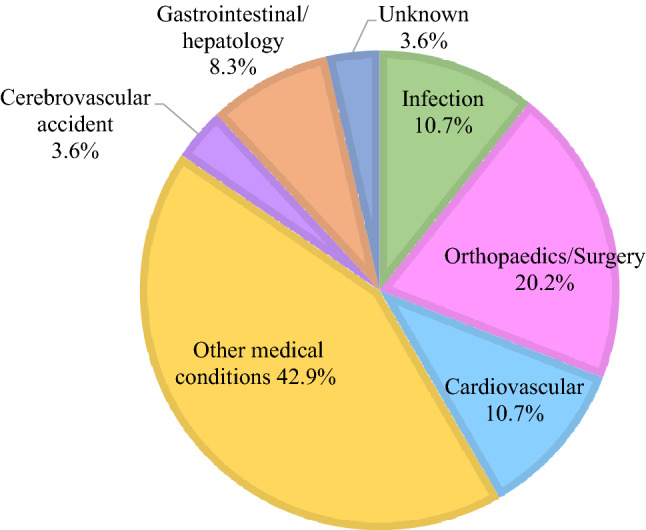
Reasons for hospital admission

All hospitalised patients were on CAPD when they developed peritonitis. The median duration (IQR) of hospital stay prior to the development of HaP was 12 (4–28) days, and the duration (IQR) of hospital stay after developing HaP was 14 (5–26) days. Five (1.1%) patients with 56.3 ± 50.4 days hospital stay had 2 episodes of HaP during the study period. Figure 2 summarised the reasons for their hospital admission.

#### Invasive procedures

Of the 84 episodes of HaP, 17 (20.2%) episodes were associated with invasive procedures. Of which, 29.4% (*n* = 5/17) were angiograms and 23.5% (*n* = 4/17) were enteroscopies, while 8 (47.1%) episodes involved a surgical procedure. Of those who had a surgical procedure, 50% (*n* = 4/8) had orthopaedic surgery, 25% (*n* = 2/8) neurosurgery, head and neck surgery, 12.5% (*n* = 1/8) abdominal surgery, and 12.5% (*n* = 1/8) had cardiothoracic surgery. Sixty-four (76.2%) episodes had no known invasive procedures, and it was unknown for 3 (3.6%) episodes. No known invasive procedures were reported in the Ca-t-HaP group.

### Laboratory characteristics

Patients with HaP had lower mean serum albumin levels at the time of diagnosis of peritonitis compared to patients with CaP (22.95 g/L vs. 25.68 g/L, p = 0.002). In addition, patients with HaP had lower median effluent white cell count and polymorphonuclear counts at the time of diagnosis compared to patients with CaP (Supplementary Table 1). There was no significant difference in median (IQR) PD effluent white cell counts in patients with culture-negative and culture-positive HaP (958 (264–3600) vs. 1710 (252–5815), *p* = 0.609). No significant differences in other laboratory characteristics were found between the HaP and Ca-t-HaP groups.

### Causative organisms

The distribution of causative organisms identified in the CaP and HaP episodes is summarised in Table 2. Overall, there were higher rates of *vancomycin-resistant enterococcus* (VRE) (2.4% vs. 0.0%, *p* = 0.009) and *Pseudomonas spp*. (9.5% vs. 3.7%, *p* = 0.020) in the HaP group than the CaP group. Compared to the HaP group, *Serratia spp.* (18.2% vs. 0.0%, *p* = 0.012) and *Candida spp.* (18.2% vs. 1.2%, *p* = 0.035) were more commonly isolated in the Ca-t-HaP group.

**Table 2:** Microbiology of the CaP and HaP groups

Type of organism	Peritonitis episodes (*n* = 893)^*^		
	CaP (*n* = 809)	HaP (*n* = 84)	*P*^b^
Culture negative, *n* (%)	128 (15.8%)	15 (17.9%)	0.639
Gram-positive organisms, *n* (%)^a^	385 (47.6%)	36 (42.9%)	0.424
* Methicillin-susceptible Staphyloccocus aureus*	54 (6.7%)	2 (2.4%)	0.156
* Methicillin-resistant Staphylococcus aureus*	8 (1.0%)	2 (2.4%)	0.241
* Coagulase-negative staphylococcus*	201 (24.8%)	13 (15.5%)	0.060
* Streptococcus spp.*	76 (9.4%)	8 (9.5%)	1.000
* Enterococcus spp. (Enterococcus faecalis/faecium)*	18 (2.2%)	3 (3.6%)	0.438
* Vancomycin-resistant Enterococcus*	0 (0.0%)	2 (2.4%)	0.009
* Diphtheroids (Corynebacteria)*	10 (1.2%)	3 (3.6%)	0.115
Other Gram-positive organisms	18 (2.2%)	3 (3.6%)	0.438
Gram-negative organisms, *n* (%)	204 (25.2%)	22 (26.2%)	0.845
* Escherichia coli*	60 (7.4%)	8 (9.5%)	0.514
* Pseudomonas spp.*	30 (3.7%)	8 (9.5%)	0.020
* Klebsiella spp.*	27 (3.3%)	2 (2.4%)	1.000
* Acinetobacter spp.*	14 (1.7%)	1 (1.2%)	1.000
* Serratia spp.*	20 (2.5%)	0 (0.0%)	0.245
* Enterobacter spp.*	16 (2.0%)	1 (1.2%)	1.000
* Citrobacter spp.*	7 (0.9%)	1 (1.2%)	0.548
* Neisseria spp.*	2 (0.2%)	0 (0.0%)	1.000
* Stenotrophomonas maltophilia*	4 (0.5%)	0 (0.0%)	1.000
Other Gram-negative organisms	24 (3.0%)	1 (1.2%)	0.719
Polymicrobial, *n* (%)	58 (7.2%)	7 (8.3%)	0.660
Candida, *n* (%)	22 (2.7%)	1 (1.2%)	0.715
Other fungal/yeast, *n* (%)	5 (0.6%)	1 (1.2%)	0.448
Other organisms, *n* (%)	4 (0.5%)	1 (1.2%)	0.390
No information, *n* (%)	3 (0.4%)	1 (1.2%)	0.327

*Not inclusive of the episodes in the community-then-hospital-acquired peritonitis (Ca-t-HaP) group (*n* = 11)

^a^May not add up to 100% due to rounding

^b^Chi-square test or Fisher exact test, as appropriate

### Outcomes

#### Primary outcomes

Compared with CaP, HaP episodes had significantly lower odds of complete cure (39.3% vs. 61.7%; OR = 0.40; 95% CI = 0.254–0.637). Refractory peritonitis was significantly higher in the HaP group (39.3% vs. 16.4%; OR = 3.289; 95% CI = 2.044–5.292). In addition, higher odds of refractory peritonitis (81.8% vs. 39.3%; OR = 6.955; 95% CI = 1.413–34.223) were observed in the Ca-t-HaP group compared to the HaP group. The clinical outcomes for the CaP and HaP groups are summarised in Table 3.

**Table 3:** Primary and secondary outcomes of CaP and HaP episodes

	No. of episodes (*n* = 893)^a^			
	CaP (*n* = 809)	HaP (*n* = 84)	*P*^d^	OR (95% CI)
Primary outcome, *n* (%)^b^				
Complete cure	499 (61.7%)	33 (39.3%)	< 0.001	0.402 (0.254–0.637)
Refractory	133 (16.4%)	33 (39.3%)	< 0.001	3.289 (2.044–5.292)
Recurrence	82 (10.1%)	4 (4.8%)	0.123	0.443 (0.158–1.241)
Relapse	59 (7.3%)	6 (7.1%)	1.000	0.978 (0.409–2.338)
No information	17 (2.1%)	1 (1.2%)	1.000	0.561 (0.074–4.271)
Secondary outcome, *n* (%)^b^				
PD-catheter removal	133 (16.4%)	21 (25%)	0.048	1.694 (1.000–2.872)
All-cause mortality^c^ within 30 days of peritonitis	27 (3.3%)	24 (28.6%)	< 0.001	11.585 (6.299–21.307)

*CaP* community-acquired peritonitis, *HaP* hospital-acquired peritonitis, *PD* peritoneal dialysis

^a^Not inclusive of the episodes in the community-then-hospital-acquired peritonitis (Ca-t-HaP) group (*n* = 11)

^b^May not add up to 100% due to death that occurred during the treatment of peritonitis

^**c**^Death due to other medical conditions and PD-associated peritonitis

^d^Chi-square test or Fisher-exact test, as appropriate

#### Secondary outcomes

Compared to the CaP group, PD-catheter removal (25% vs. 16.4%, OR = 1.694; 95% CI = 1.000–2.872) and all-cause mortality within 30 days of peritonitis diagnosis (28.6% vs. 3.3%; OR = 11.585; 95% CI = 6.299–21.307) were significantly higher in the HaP group. In addition, there were higher odds of PD-catheter removal (81.8% vs. 25%; OR = 13.500; 95% CI = 2.699–67.525) observed in the Ca-t-HaP compared to the HaP group.

### Predictors of PD-associated peritonitis outcomes in HaP and CaP groups

#### Complete cure

After adjusting the GEE model for other variables, a higher serum albumin level (adjusted odds ratio [AOR] = 1.062; 95% CI = 1.029–1.095) was associated with higher odds of complete cure. The causative organisms of the peritonitis episodes associated with the highest odds of complete cure were culture-negative peritonitis (AOR = 4.843; 95% CI = 2.263–10.364). There was no statistical difference in the odds of complete cure between the HaP and CaP episodes (Table 4).

**Table 4:** Generalised estimating equation analysis of predictors for complete cure, all-cause mortality, refractory peritonitis and PD-catheter removal in CaP and HaP groups

Variables	Complete cure		All-cause mortality		Refractory peritonitis		PD-catheter removal	
	Adjusted value		Adjusted value		Adjusted value		Adjusted value	
	AOR	*p*	AOR	*p*	AOR	*p*	AOR	*p*
HaP vs. CaP	0.710 (0.397–1.269)	0.248	6.360 (2.800–14.443)	< 0.001	1.632 (0.897–2.968)	0.109	0.994 (0.546–1.812)	0.985
Age (years)	0.994 (0.978–1.010)	0.436	1.045 (1.007–1.084)	0.020	*	*	*	*
Duration of PD (months)	*	*	–	–	1.005 (0.998–1.012)	0.145	*	*
Coronary artery disease	*	*	2.424 (1.086–5.409)	0.031	*	*	*	*
Cerebrovascular disease	*	*	0.903 (0.307–2.661)	0.853	0.678 (0.346–1.326)	0.256	0.545 (0.265–1.120)	0.099
Diabetes mellitus	0.947 (0.613–1.462)	0.804	*	*	*	*	*	*
Peripheral vascular disease	*	*	1.002 (0.247–4.069)	0.997	*	*	*	*
Chronic lung disease	0.692 (0.353–1.358)	0.285	*	*	*	*	*	*
Serum albumin level	1.062 (1.029–1.095)	< 0.001	0.943 (0.891–0.999)	0.044	0.966 (0.937–0.996)	0.028	0.977 (0.948–1.007)	0.130
Culture-negative	4.843 (2.263–10.364)	< 0.001	*	*	0.257 (0.123–0.539)	< 0.001	0.295 (0.136–0.640)	0.002
Polymicrobial	1.084 (0.473–2.485)	0.849	*	*	1.643 (0.814–3.315)	0.166	2.103 (1.056–4.188)	0.035
*Enterococcus spp*. (*Enterococcus faecalis/faecium*)	0.852 (0.196–3.698)	0.831	4.177 (0.744–23.465)	0.104	*	*	*	*
MSSA	3.248 (1.268–8.318)	0.014	*	*	0.330 (0.109–0.998)	0.050	0.387 (0.129–1.160)	0.090
* Streptococcus spp.*	4.821 (2.034–11.423)	< 0.001	0.178 (0.027–1.185)	0.074	0.170 (0.060–0.482)	< 0.001	0.151 (0.044–0.510)	0.002
CNSS	3.377 (1.785–6.388)	< 0.001	0.169 (0.039–0.740)	0.018	0.176 (0.085–0.363)	< 0.001	0.219 (0.107–0.451)	< 0.001
Other gram-positive	*	*	*	*	1.103 (0.345–3.521)	0.869	1.449 (0.417–5.032)	0.559
* E.coli*	1.101 (0.487–2.489)	0.817	*	*	*	*	*	*
* Klebsiella spp.*	1.638 (0.527–5.091)	0.394	*	*	*	*	*	*
* Pseudomonas spp.*	0.279 (0.073–1.070)	0.063	*	*	6.601 (2.592–16.810)	< 0.001	8.429 (3.159–22.494)	< 0.001
* Serratia spp.*	0.323 (0.058–1.802)	0.197	*	*	1.005 (0.998–1.012)	0.145	3.584 (0.938–13.697)	0.062

*AOR* adjusted odds ratio, *CaP* community-acquired peritonitis, *CI* confidence interval, *HaP* hospital-acquired peritonitis, *OR* odds ratio, *PD* peritoneal dialysis, *CNSS* Coagulase-negative *Staphylococcus*; *E.coli* *Escherichia coli*, *MRSA* Methicillin-resistant *Staphylococcus aereus*, *MSSA* Methicillin-susceptible *Staphylococcus aureus*

*Excluded from the analysis as the p-value exceeded 0.2 in the univariate analysis (Supplementary Table 2)

#### All-cause mortality

Using the adjusted GEE model, lower serum albumin level (AOR = 0.943; 95% CI = 0.891–0.999), the presence of HaP vs. CaP (AOR = 6.360; 95% CI = 2.800–14.443) and older age (AOR = 1.045; 95% CI = 1.007–1.084) were associated with higher odds of all-cause mortality. Additionally, peritonitis caused by coagulase-negative *Staphylococcus* (CNSS) (AOR = 0.169; 95% CI = 0.039–0.740) was associated with lower odds of all-cause mortality (Table 4).

#### Refractory peritonitis and PD-catheter removal

In the adjusted GEE model, peritonitis caused by *Pseudomonas spp.* was associated with higher odds of refractory peritonitis and PD-catheter removal. Lower serum albumin was associated with higher odds of refractory peritonitis but showed no statistical difference in the odds of PD-catheter removal. On the other hand, polymicrobial peritonitis was associated with higher odds of PD-catheter removal. However, culture-negative peritonitis, *Streptococcus spp*. and CNSS were associated with lower odds of refractory peritonitis and PD-catheter removal (Table 4). There was no statistical difference in the odds of refractory peritonitis and PD-catheter removal between the HaP and CaP episodes (Table 4).

## Discussion

In this study, we found that the serum albumin level and initial effluent white cell count, particularly polymorphonuclear counts, at the time of diagnosis were significantly lower in the HaP group. Secondly, compared with the CaP group, *Pseudomonas spp.* and VRE were more prevalent in the HaP group. Thirdly, episodes in the HaP group had poorer outcomes with lower rates of complete cure and higher rates of refractory peritonitis, PD-catheter removal and all-cause mortality within 30 days of peritonitis diagnosis as compared to the CaP group. Next, there was an underlying difference between the HaP and CaP groups, mainly the complete cure, all-cause mortality, refractory peritonitis, and PD-catheter removal rates, which could be explained by underlying predictors such as types of causative organisms and serum albumin level. Overall, while the rate of HaP in our study was 9.3% over the 10-year study period, which is relatively lower compared to the rate of 5% over 1 year demonstrated by Troidle et al*.* [6], it is similar to the peritonitis rate shown by Szeto et al. [10] This may be reflective of an improved diagnostic and treatment approach to PD-associated peritonitis over the years.

Our study revealed that patients in the HaP group had lower mean serum albumin levels at the time of peritonitis diagnosis compared to the CaP group (22.95 g/L vs. 25.76 g/L, respectively). Consistent with previous studies [6, 11, 12], our findings demonstrated lower serum albumin levels as a predictor for all-cause mortality and refractory peritonitis in PD patients. In contrast, a higher serum albumin level was associated with higher odds of complete cure. Hypoalbuminaemia is a common problem among hospitalised patients [13], and the aetiology in hospitalised patients on PD could be multifactorial. Hypoalbuminaemia may have resulted from malnourishment due to insufficient nutrient intake caused by reduced appetite [5], protein catabolism and losses while receiving PD during PD-associated peritonitis [14]. In addition to the usual dietary interventions to prevent and treat malnutrition during hospitalisation, the use of amino-acid-based PD solutions (e.g. Nutrineal^®^, Baxter Healthcare) could be a promising intervention to improve nutritional parameters in hypoalbuminaemic patients on PD by stimulating protein anabolism [15, 16]. However, the use of these solutions to date has not been shown to improve peritonitis outcomes [17]. Nonetheless, the prevention and treatment of malnutrition in hospitalised PD patients can be optimised in collaboration with dieticians.

Moreover, we also observed that episodes in the HaP group had significantly lower effluent white cell and polymorphonuclear cell counts at the time of diagnosis of peritonitis compared with the CaP group, indicating that HaP was potentially detected earlier than CaP as patients were hospitalised. This allows prompt sampling of the dialysate at the earliest suspicion of peritonitis. PD effluent white cell counts can also be influenced by the virulence of the infecting organism, the length of PD dwell, prior antibiotic use and the host’s response to the infection. In our study, patients with HaP had a higher proportion of infections from *Pseudomonas spp.* and VRE, indicating infections from more virulent organisms in patients with HaP. Furthermore, PD effluent white cell counts of patients with culture-negative HaP (a possible indicator of prior antibiotic use) were not significantly different from those with culture-positive HaP. Another potential reason for patients with HaP having lower PD effluent white cell counts could be their poorer host response, since patients with HaP also had lower serum albumin levels compared to patients with CaP.

In line with the study reported by Szeto et al. [10], we found that patients with HaP had poorer outcomes compared to those with CaP, despite the lower initial effluent white cell counts. This is consistent with the study reported by Chow et al*.* [18], in which the authors proposed that the effluent cell counts on Day 3 (while on antibiotic treatment), as opposed to Day 1, maybe a better predictor of treatment outcomes. However, it is also noteworthy that the study recruited patients treated in the outpatient setting. Although a delay in antibiotic commencement for the treatment of PD-associated peritonitis is associated with poorer outcomes, we could not accurately analyse the time from the onset of peritonitis to the initiation of antibiotic treatment due to the retrospective nature of this study. Of note, the findings from the Presentation and the Time of Initial Administration of Antibiotics With Outcomes of Peritonitis (PROMPT) study suggested that for each hour of delay of antibiotics administration, the risk of PD failure and mortality increased by 5.5% (OR: 1.055; 95% CI 1.005–1.109; *p* = 0.032) [19]. Despite the potential early commencement of antibiotics in patients with HaP compared to those with CaP, the HaP group had poorer outcomes. Contrary to the study by Chow et al. [18], our findings observed that the poorer outcomes of peritonitis in the HaP group were likely a result of the type of causative microorganisms and the poorer nutritional status of patients at the time of developing peritonitis. Moreover, the lower dialysate white cell count at diagnosis may be reflective of an early diagnosis of HaP rather than the lower severity of the infection at the time of diagnosis. Whilst most of the invasive procedures in the HaP group were angiograms, there was no evidence to suggest that these procedures were causally associated with the development of PD-associated peritonitis.

We found some variation in the distribution of the causative microorganisms in this study compared to previous reports [6, 10] between the HaP and CaP groups. Troidle et al*.* [6] identified higher rates of *Staphylococcal spp*., *Enterococcus spp*. and gram-negative organisms in the HaP group, while Szeto et al*.* [10]. found a higher prevalence of culture-negative peritonitis in the HaP group [10], possibly due to problems handling PD effluent samples and recent antibiotic therapy during hospital admission [20]. Conversely, our study observed higher rates of *Pseudomonas spp.* and VRE in the HaP group. The potential reasons for the higher rates of *Pseudomonas spp.* and VRE in our study may be due to variations in the microbiological spectrum of HaP that may differ across different regions and time periods. Furthermore, a longer length of hospital stay prior to the development of peritonitis in our patients (median hospital stay was 12 days) may have exposed them to more resistant strains from colonisation or antibiotic exposure during hospitalisation. Previous studies have also highlighted that recent antibiotic use [21–23] alters the normal gastrointestinal flora leading to the transmural migration of the organisms to the peritoneal cavity [3, 22]. This, along with the length of hospital stay [24, 25] are significant risk factors for *Pseudomonas spp.* and *Enterococcus spp.* infections in hospitalised patients and have been associated with poorer outcomes in PD patients [26, 27]. Nevertheless, our findings are consistent with those by Szeto et al. [10], which demonstrated similar rates of HaP and poorer outcomes in the HaP compared to the CaP group.

Interestingly, we found that *Serratia spp.* and *Candida spp.* were more commonly isolated in the Ca-t-HaP group and were associated with significantly higher rates of PD-catheter removal than in the HaP group (81.8% vs. 25%). Possible explanations for these findings include several potential risk factors which have been highlighted in other studies, including: prolonged duration of hospitalisation [28], recent use of broad-spectrum antibiotics [29] for the treatment of CaP, suboptimal glycaemic control in PD patients with diabetes mellitus during acute infection [30], impaired immune response secondary to renal failure [31], and touch contamination of the PD exit-site by untrained nursing staff and acutely unwell patients [32].

Consistent with previous studies [8, 33–36], our findings have also revealed that the gram-positive organism (i.e., CNSS) and culture-negative peritonitis were predictors associated with higher odds of complete cure and lower odds of all-cause mortality, refractory peritonitis, and PD-catheter removal. It is well-recognised that culture-negative peritonitis episodes that improve promptly with antibiotics are likely caused by gram-positive organisms [9] and have good outcomes. In addition, peritonitis caused by CNS represents a heterogeneous group, ranging from low, medium, or even high virulence potentials with well-recognised adhesive properties (that can lead to colonisation of PD catheter with biofilm formation) as well as high rates of methicillin resistance [9, 37]. Whilst most CNS are easy to grow in the laboratory, a small population of CNS may be fastidious and present as ‘small colony variants (SCVs)’ [37]. Furthermore, CNS (especially the SCVs) grown on PD fluid cultures may be considered by some laboratories as contaminants and be reported as ‘no growth’. This was not the case in our study, as our laboratories routinely report all CNS growth in PD fluids.

Although Mujais [35] did not compare the microbiology and outcomes of peritonitis between HaP and CaP groups, our findings revealed similar peritonitis outcomes as Mujais [35]. Consistent with Mujais, our study also observed clustering of peritonitis episodes, with 103 (21.8%) patients having three or more episodes of peritonitis during the study period. Of these, only 5 (1.1%) patients had 2 episodes of HaP during the study period and had hospital stays of 56.3 ± 50.4 days. The low frequency of clustering of HaP may be due to fewer numbers and/or short periods of hospitalisation in individual patients of PD. Future studies are warranted to examine clinical characteristics and organisms that predispose to or are associated with multiple episodes or clustering of peritonitis. Nonetheless, our findings along with Mujais [35] and Szeto et al. [10], emphasise the importance of determining the underlying cause of HaP and organism-specific rates as a measure of continuous quality improvement and considering unit-specific empiric antibiotic treatment based on the spectrum of organisms causing HaP and their sensitivity patterns.

Peritonitis caused by gram-positive organisms is commonly caused by touch contamination [9] and causes relatively less severe peritonitis with considerably better outcomes than gram-negative organisms [33, 34]. Although comparing peritonitis risks or rates between patients on CAPD and APD was not one of the aims of this study, we did observe that more than 90% of patients who developed an episode of peritonitis during the study period were on CAPD. A lower risk of peritonitis in patients on APD compared to those on CAPD is thought to occur due to fewer manual connections and the potential for touch contamination in patients on APD. However, this was not a factor in the HaP group in our study, as all hospitalised patients were on CAPD. Considering that PD patients may be admitted to the general wards, nurses unfamiliar with PD may incidentally introduce touch contamination while performing PD exchanges. Furthermore, as patients on PD may be admitted to different wards of the hospital based on their admission diagnosis, the nurses may not be trained to perform PD exchanges. It is also challenging to train, retrain and conduct competency assessments on all the nurses in each ward on a regular basis. Therefore, admitting patients on PD in areas where nursing staff are trained to conduct PD exchanges, ensuring that during-hours and after-hours nurse educators are trained and competent in PD, can be important to reduce the risk of acquiring HaP. In addition, other approaches include (i) allowing patients (or their carers) to conduct PD exchanges themselves while they are hospitalised, (ii) putting patients on APD with a cycler to reduce touch contamination by untrained or inexperienced nursing staff, and (iii) omitting a few PD exchanges, if safe, until a trained PD nurse is available can be considered to prevent HaP.

There are a few limitations of our study. Firstly, the small numbers of some causative organisms in the HaP group may have resulted in wide confidence intervals. Therefore, the results of this study have to be interpreted with caution. Secondly, incomplete documentation due to the retrospective nature of this study cannot be excluded. Thus, we may not have been able to identify other potential predictors that could affect the outcome of PD-associated peritonitis. Thirdly, our study did not capture the administration of prophylactic antibiotics prior to the invasive procedures in the HaP group. Fourthly, due to the retrospective nature of the study, information on the PD effluent drainage prior to the invasive gastrointestinal procedures was unavailable. Furthermore, there is a likelihood of practice variations in the use of prophylactic antibiotics prior to invasive gastrointestinal procedures done on patients undergoing PD. Practice evolution in managing and preventing peritonitis could have also occurred. Nonetheless, to our knowledge, this is the first study on HaP across four teaching hospitals in Australia with patients from diverse ethnic backgrounds. Given the large sample size of this study and a good distribution of CaP and HaP episodes across the entire study period, our findings can be generalised to other centres in Australia as the treatment of PD-associated peritonitis in most centres is based on protocols derived from the ISPD peritonitis guidelines. This study also provides important insight into developing and implementing an antibiotic stewardship programme for HaP to guide the appropriate use of empirical antibiotics and monitor centre-specific resistance patterns.

In summary, patients with HaP are more likely to be malnourished and develop peritonitis due to *Pseudomonas spp.* and VRE. Furthermore, despite the lower PD effluent cell count at the time of diagnosis and the potential prompt commencement of antibiotic treatment, HaP was associated with poorer outcomes. Therefore, potential interventions to improve the outcomes of patients with HaP include (i) effective antimicrobial stewardship and avoiding unnecessary empirical antibiotics in hospitalised PD patients, (ii) early identification and commencement of antibiotic treatment of every episode of HaP, (iii) modifying choice of empirical antibiotic regimens based on the unit-specific bacterial isolates in patients with HaP and (iv) use of antifungal prophylaxis during the antibiotic course to prevent fungal peritonitis. Furthermore, proactive identification of refractory peritonitis with early removal of PD catheter, as recommended by the 2022 ISPD peritonitis guidelines [9], may reduce mortality rates in patients with HaP. Finally, PD centres could also consider performing root cause analysis for every episode of HaP, monitoring and benchmarking HaP rates in each PD centre, at least annually, for continuous quality improvement. Further studies are also warranted to identify the risk factors of HaP to evaluate different prophylactic and therapeutic approaches in the HaP setting.

### Supplementary Information

Below is the link to the electronic supplementary material.

Supplementary file1 (DOCX 19 KB)
