# Supplementary material for: Characteristics and outcomes of hospital-acquired and community-acquired peritonitis in patients on peritoneal dialysis: a retrospective cohort study
Source: J Nephrol. 2023 Mar 13;36(7):1877–88. doi: 10.1007/s40620-023-01597-w (PMC10543707; doi:10.1007/s40620-023-01597-w)
Supplement: Supplementary file 1 — Supplementary file1 (DOCX 19 KB) [file 40620_2023_1597_MOESM1_ESM.docx]

**SUPPLEMENTARY MATERIAL**

**Characteristics and outcomes of hospital-acquired and community-acquired peritonitis in patients on peritoneal dialysis: A retrospective cohort study**

**Chau Wei LING ^1^, Kamal SUD ^1, 2, 3^, Gregory M. PETERSON ^4,5^, Judith FETHNEY ^6^, Connie VAN ^1^, Rahul P. PATEL^4^, Syed Tabish Razi ZAIDI ^7^, Ronald L. CASTELINO ^1, 8^**

1) Faculty of Medicine and Health, The University of Sydney, New South Wales, Australia

2) Department of Renal Medicine, Nepean, Blacktown and Westmead Hospitals, Sydney, New South Wales, Australia

3) Peritoneal Dialysis Unit, Regional Dialysis Centre, Blacktown Hospital, Sydney, New South Wales, Australia

4) School of Pharmacy and Pharmacology, University of Tasmania, Hobart, Tasmania, Australia

5) Faculty of Health, University of Canberra, Bruce, Australian Capital Territory, Australia

6) Faculty of Medicine and Health, Susan Wakil School of Nursing and Midwifery, The University of Sydney

7) Professional Services Unit, HPS Pharmacies, EBOS Group, Docklands, Victoria, Australia

8) Department of Pharmacy, Blacktown Hospital, Blacktown, New South Wales, Australia

**Corresponding author**

Chau Wei LING

Faculty of Medicine and Health, The University of Sydney, NSW 2006 Australia

Email: [clin6270@uni.sydney.edu.au](mailto:clin6270@uni.sydney.edu.au)

**Supplementary Table 1**- Laboratory characteristics of peritonitis episodes between HaP and CaP

| **Laboratory characteristics** | **CaP**  **(n=809)** | **HaP**  **(n=84)** | | ***p*** |
| --- | --- | --- | --- | --- |
| Serum albumin level (g/L), mean ± SD | 25.76 ± 7.29 | 22.95 ± 6.76 | 0.002 ^a^ | |
| **Dialysate effluent white cell count (cells/mm^3^)** | | | | |
| Leucocytes, median (IQR) | 3183.50 (900.00-10350.00) | 1236.00 (265.25-5386.50) | <0.001^b^ | |
| Polymorphs, median (IQR) | 2800.00 (656.0-9540.00) | 1037.00 (180.00- 4954.50) | <0.001^b^ | |
| Mononuclear, median (IQR) | 219.00 (67.00-776.25) | 162.00 (53.50-396.00) | 0.140 ^b^ | |

^a^ Student t-test

^b^ Mann-Whitney test

CaP= Community-acquired peritonitis; HaP= Hospital-acquired peritonitis; IQR= Interquartile range; SD= Standard deviation

**Supplementary Table 2**: Generalised estimating equation analysis of predictors for complete cure, all-cause mortality, refractory and PD catheter removal in CaP and HaP groups^: Univariate analysis

|  | **Complete cure** | | **All-cause mortality** | | **Refractory peritonitis** | | **PD-catheter removal** | | |
| --- | --- | --- | --- | --- | --- | --- | --- | --- | --- |
| **Variables** | **Unadjusted value** | | **Unadjusted value** | | **Unadjusted value** | | **Unadjusted value** | | |
|  | **OR (95 % CI)** | ***p*** | **OR (95 % CI)** | ***p*** | **OR (95 % CI)** | ***p*** | | **OR (95 % CI)** | ***p*** |
| HaP vs. CaP | 0.402 (0.250-0.646) | <0.001 | 11.585 (6.066-22.126) | <0.001 | 3.289 (2.002-5.402) | <0.001 | | 1.694 (1.004-2.858) | 0.048 |
| Age (years) | 0.987 (0.976-0.998) | 0.023 | 1.067 (1.033-1.102) | <0.001 | 1.005 (0.993-1.018) | 0.410 | | 1.000 (0.987-1.012) | 0.960 |
| Duration of PD (months) | 1.000 (0.995-1.004) | 0.923 | 1.004 (0.997-1.011) | 0.219 | 1.003 (0.999-1.008) | 0.153 | | 1.002 (0.998-1.007) | 0.300 |
| Female gender | 0.909 (0.670-1.234) | 0.542 | 0.981 (0.534-1.802) | 0.951 | 1.103 (0.787-1.546) | 0.568 | | 1.032 (0.731-1.457) | 0.859 |
| Coronary artery disease | 1.035 (0.770-1.390) | 0.820 | 1.993 (1.085-3.662) | 0.026 | 0.978 (0.697-1.373) | 0.898 | | 0.871 (0.611-1.242) | 0.447 |
| Diabetes mellitus | 0.789 (0.588-1.059) | 0.114 | 1.063 (0.577-1.957) | 0.845 | 1.060 (0.761-1.478) | 0.729 | | 1.134 (0.807-1.594) | 0.468 |
| Cerebrovascular disease | 0.987 (0.675-1.444) | 0.947 | 1.847 (0.844-4.046) | 0.125 | 0.642 (0.350-1.176) | 0.151 | | 0.510 (0.259-1.002) | 0.051 |
| Chronic lung disease | 0.695 (0.455-1.061) | 0.092 | 1.320 (0.566-3.080) | 0.521 | 1.291 (0.807-2.063) | 0.286 | | 1.237 (0.758-2.021) | 0.395 |
| Peripheral vascular disease | 1.104 (0.592-2.060) | 0.755 | 2.106 (0.780-5.689) | 0.142 | 1.196 (0.580-2.465) | 0.627 | | 1.137 (0.534-2.422) | 0.740 |
| Serum albumin level | 1.064 (1.035-1.094) | <0.001 | 0.924 (0.878-0.972) | 0.002 | 0.966 (0.940-0.992) | 0.012 | | 0.978 (0.953-1.005) | 0.108 |
| Culture-negative | 1.927 (1.250-2.970) | 0.003 | 0.686 (0.286-1.647) | 0.399 | 0.254 (0.128-0.501) | <0.001 | | 0.245 (0.120-0.502) | <0.001 |
| Polymicrobial | 0.595 (0.358-0.988) | 0.045 | 1.769 (0.724-4.324) | 0.211 | 2.623 (1.547-4.449) | <0.001 | | 3.143 (1.850-5.340) | <0.001 |
| ***Enterococcus spp*. (*Enterococcus faecalis/faecium*)** | 0.500 (0.202-1.240) | 0.135 | 4.130 (1.305-13.075) | 0.016 | 0.725 (0.206-2.554) | 0.617 | | 0.499 (0.113-2.206) | 0.359 |
| MRSA | 1.018 (0.285-3.638) | 0.978 | 1.851 (0.230-14.901) | 0.563 | 1.096 (0.232-5.184) | 0.908 | | 0.530 (0.067-4.201) | 0.548 |
| MSSA | 2.940 (1.519-5.689) | 0.001 | 0.930 (0.283-3.055) | 0.905 | 0.321 (0.114-0.903) | 0.031 | | 0.352 (0.125-0.993) | 0.048 |
| *Streptococcus spp.* | 3.438 (1.903-6.209) | <0.001 | 0.183 (0.026-1.295) | 0.089 | 0.200 (0.072-0.553) | 0.002 | | 0.161 (0.050-0.520) | 0.002 |
| CNSS | 1.675 (1.186-2.367) | 0.003 | 0.406 (0.170-0.972) | 0.043 | 0.203 (0.112-0.366) | <0.001 | | 0.247 (0.140-0.435) | <0.001 |
| Other gram-positive | 0.741 (0.296-1.857) | 0.523 | 1.768 (0.386-8.099) | 0.463 | 1.780 (0.754-4.200) | 0.188 | | 1.957 (0.829-4.621) | 0.126 |
| *E.coli* | 0.596 (0.341-1.041) | 0.069 | 1.707 (0.691-4.217) | 0.246 | 1.414 (0.818-2.445) | 0.215 | | 1.170 (0.648-2.110) | 0.603 |
| *Pseudomonas spp.* | 0.197 (0.09-0.429) | <0.001 | 1.441 (0.433-4.793) | 0.551 | 8.608 (4.607-16.081) | <0.001 | | 7.531 (4.055-13.987) | <0.001 |
| *Diphtheroids* | 1.087 (0.365-3.237) | 0.881 | * | * | 0.794 (0.167-3.770) | 0.771 | | 0.871 (0.183-4.137) | 0.862 |
| *Klebsiella spp.* | 0.467 (0.217-1.005) | 0.052 | 1.962 (0.582-6.616) | 0.277 | 1.148 (0.454-2.902) | 0.771 | | 0.762 (0.257-2.255) | 0.623 |
| *Acinetobacter spp.* | 0.870 (0.298-2.541) | 0.799 | * | * | 0.621 (0.138-2.786) | 0.534 | | 0.681 (0.152-3.064) | 0.617 |
| *Citrobacter spp.* | 1.132 (0.242-5.308) | 0.875 | * | * | 0.623 (0.073-5.306) | 0.665 | | 0.683 (0.080-5.819) | 0.728 |
| *Enterobacter spp.* | 0.759 (0.276-2.091) | 0.594 | 2.250 (0.545-9.300) | 0.263 | 0.937 (0.263-3.345) | 0.921 | | 0.635 (0.142-2.838) | 0.552 |
| Other gram-negative | 1.457 (0.616-3.444) | 0.392 | 1.453 (0.331-6.376) | 0.620 | 1.397 (0.546-3.575) | 0.485 | | 1.206 (0.443-3.283) | 0.713 |
| *Serratia spp.* | 0.219 (0.078-0.618) | 0.004 | * | * | 3.017 (1.200-7.586) | 0.019 | | 3.320 (1.318-8.360) | 0.011 |

**^^^** Some organisms (*Neiserria spp. Stenotrophomonas maltophilia, Candida*, other fungal/yeast, other organisms, *Vancomycin-resistant* enterococcus [VRE]) were excluded from the analysis due to the small number of peritonitis episodes caused by these organisms

* Unable to compute due to small number of peritonitis episodes caused by this organism

AOR= adjusted odds ratio; CaP= community-acquired peritonitis; CI = confidence interval; HaP= hospital-acquired peritonitis; OR= odds ratio; PD= peritoneal dialysis

CNSS= Coagulase-negative *Staphylococcus*; E.coli= *Escherichia coli*; MRSA= Methicillin-resistant *Staphylococcus aureus*; MSSA= Methicillin-susceptible *Staphyloccocus aureus*
